# Postpartum Discharge Readiness and Preparation: A Scoping Review

**DOI:** 10.3390/healthcare14213827

**Published:** 2026-11-09

**Authors:** Petronella Lunda, Lydia Boampong Owusu, Barbara Zych, Małgorzata Nagórska

**Affiliations:** 1 School of Nursing, Faculty of Health Sciences, North-West University, Potchefstroom 2531, South Africa; 2 School of Nursing and Midwifery, Kwame Nkrumah University of Science and Technology, Kumasi 00233, Ghana; 3 Faculty of Health Sciences and Psychology, Collegium Medicum, University of Rzeszow, 35-959 Rzeszow, Poland

**Keywords:** discharge readiness, postnatal self-care, postpartum mothers, scoping review

## Abstract

**Highlights:**

**What are the main findings?**

- Postpartum education and structured interventions have been associated with improved maternal outcomes, including discharge readiness, self-confidence, and neonatal care knowledge, attitudes, and practices.
- Discharge experiences and readiness are also shaped by broader health system and contextual factors such as continuity of care, maternal understanding of postpartum care, and facility policies.

**What are the implications of the main findings?**

- Strengthening structured postpartum education in maternity services can improve maternal competence, confidence in newborn care, and safer transitions from hospital to home.
- Health facilities should address gaps in postnatal care, such as inconsistent discharge practices and limited patient education, to improve continuity of care and ensure women are prepared for early discharge and postnatal self-care.

**Abstract:**

**Background:** Discharge readiness is an important aspect of postpartum care and has been linked to maternal confidence, safety, and outcomes after hospital discharge. While guidelines support timely discharge for low-risk mothers, there is variation in how readiness is assessed and experienced. The purpose of this scoping review was to map the evidence on postpartum discharge preparation and transitional support, clarify how discharge readiness is understood, and identify gaps to guide future research and practice. **Methods:** A systematic search was conducted in PubMed, EBSCOhost, Scopus, and Web of Science for literature published between January 2014 and October 2025. Eligible studies focused on the readiness of postpartum mothers for the transition from hospital to home. **Results:** Six studies were included in the synthesis. They indicate that readiness for discharge and the transition to home-based postpartum care are associated with educational, psychosocial, clinical, and health-system factors. **Conclusions:** Discharge readiness appears to be a complex, multidimensional process. It is linked to structured education, maternal confidence and caregiving skills, woman-centred support, attention to physical and mental health, and health-system processes that support continuous and consistent care. Further research using stronger designs is needed to clarify whether these factors influence readiness and post-discharge outcomes.

## 1. Introduction

The postpartum period, commonly defined as the first six weeks following childbirth, is a critical phase of maternal physical recovery, emotional adjustment, and transition to newborn care [1]. Although maternity facilities provide essential care, the move from hospital to home can be difficult. Women frequently experience emotional isolation, limited practical help, and difficulty navigating healthcare systems, particularly in underserved populations [2,3]. Inadequate support networks increase vulnerability to postpartum depression and anxiety [4], limited assistance with newborn care and household tasks compounds recovery challenges [5], and limited health literacy or financial resources can delay access to follow-up care [6]. Effective postpartum education and discharge preparation are therefore essential to support maternal confidence, promote safe home-based care, and ensure continuity of care beyond the facility.

Discharge readiness encompasses a woman’s preparedness to leave the facility, manage her recovery, recognize her health needs, care for her newborn, and access healthcare when necessary. Achieving it requires more than routine discharge instructions. Women need clear, comprehensive, individualized, and understandable information on physical recovery, emotional well-being, newborn care, and available support; they also need opportunities to ask questions and demonstrate their understanding of essential care practices before discharge. Inadequate preparation, limited health literacy, insufficient practical support, and difficulty accessing follow-up services may leave women uncertain about managing care at home [2,3,6].

Postpartum complications are classified as early (within 7 days of delivery) or late (8 days to 6 weeks postpartum) [1,7]. Because early complications can arise within days of birth, mothers who are discharged soon after delivery may need to recognize warning signs and seek care promptly, often without direct professional supervision [8,9,10]. Late complications, including those affecting emotional well-being and maternal functioning, may only become evident after discharge, when mothers are managing recovery and newborn care at home [4,11,12].

This timing highlights the importance of preparing for discharge after childbirth. Postpartum education that covers danger signs, self-care, and when and where to seek help is associated with greater maternal knowledge and confidence at discharge [11]. Preparation is important because the most serious risks are time-sensitive. Excessive bleeding accounts for approximately 25% of maternal deaths worldwide [13] and may manifest after 24 h up to 12 weeks postpartum [14]. Cardiovascular, thromboembolic, and wound-related problems are more likely when monitoring is inadequate, and treatment is delayed [15].

Education can help women recognize warning signs, understand when and where to seek care, and avoid delay in seeking medical care [8,9,10]. Preparation should also address expected physical and emotional changes, hygiene, perineal or wound care, nutrition, and rest, together with information on services for women experiencing anxiety, low mood, or social isolation [4,5]. Preparing women for newborn care is another essential component of discharge education. Breastfeeding challenges such as improper latch, nipple pain, and insufficient milk supply can hinder successful breastfeeding and contribute to neonatal complications, including jaundice [13]. Lactation support and caregiver education on positioning, attachment, and feeding are key preventive measures [16]. Newborn infections remain a major contributor to morbidity and mortality, especially after discharge, and may be classified as early-onset (within the first 72 h of life) or late-onset, both of which require prompt diagnosis and treatment [17,18]. Discharge readiness can therefore be understood as a mother’s knowledge, confidence, and ability to manage her own and her newborn’s care at home. Adequate preparation before leaving the facility may support mothers’ readiness to recognize problems and access timely care during both the early and late postpartum periods.

A woman’s voice in maternal care is essential for ensuring respectful, patient-centered healthcare. When women actively participate in decision-making, they experience better health outcomes, increased satisfaction, and improved trust in healthcare providers [19]. Ensuring discharge readiness means preparing the mother to safely and competently care for herself and her newborn at home. Well-informed mothers are better able to prevent, detect, and respond to maternal and neonatal infections, improving newborn survival in the vulnerable postnatal period.

Despite the recognized importance of postpartum education and discharge preparation, the evidence on how women are prepared to leave the maternity facility, and how they are supported during the transition to home, remains dispersed across settings, study designs, and healthcare contexts. Discharge readiness is also described and measured inconsistently, which makes it difficult to compare interventions, identify effective components, or determine where practice falls short. A scoping review is well suited to this problem, as it allows the breadth of existing evidence to be mapped and key concepts clarified, and it can show where research is lacking. The purpose of this scoping review was therefore to map the evidence on postpartum discharge preparation and transitional support, clarify how discharge readiness is understood, and identify gaps to guide future research and practice.

Although discharge education and women’s participation in care are widely promoted, it is unclear how postpartum discharge readiness is defined, measured, and supported. Evidence on its components and on the interventions that improve it is scattered and inconsistent, and attention to the mother’s own well-being is limited. This scoping review aimed to map the evidence on discharge preparation practices and transitional support for postpartum women moving from maternity facilities to home-based care and to identify gaps for future research.

## 2. Materials and Methods

### 2.1. Methods

This scoping review was conducted in accordance with the methodological guidance for scoping reviews described in the Joanna Briggs Institute (JBI) Manual for Evidence Synthesis [20] and was reported according to the PRISMA Extension for Scoping Reviews (PRISMA-ScR) [21]. The review process was also informed by the framework proposed by Arksey and O’Malley [22]. The review explored, described, and mapped evidence on factors associated with postpartum discharge readiness and preparation for transition from facility-based maternity care to home-based care.

#### 2.1.1. Step 1: Review Purpose

The purpose of this scoping review was to map the evidence on postpartum discharge preparation and transitional support, clarify how discharge readiness is understood, and identify gaps to guide future research and practice.

#### 2.1.2. Step 2: Formulating the Research Question

The research question was developed using the Population, Concept, and Context (PCC) framework to define inclusion criteria and support a comprehensive literature search [20]. This framework ensures that the review question is broad yet specific enough to capture relevant literature.

The search process followed the PRISMA-ScR (Preferred Reporting Items for Systematic Reviews and Meta-Analyses Extension for Scoping Reviews) guidelines developed [21]. By integrating the PCC framework into the development of the review question, the scoping review maintained methodological rigor and transparency. Table 1 outlines the PCC elements applied in this review.

The review did not prespecify a single operational definition of discharge readiness; instead, definitions and components reported in the included studies were charted and mapped.

##### The Review Question

What evidence is available on discharge preparation practices and transitional support for postpartum women moving from maternity facilities to home-based care across diverse global settings?

- Review Process

The first author drafted the scoping review proposal, which co-reviewers approved. The protocol was submitted to the institutional research ethics review board by the first author. After ethics approval, the protocol was registered on OSF [23] before starting the literature search. (Supplementary File S1: Review process).

#### 2.1.3. Step 3: Study Selection

According to Arksey and O’Malley, the third step in a scoping review is study selection [22]. We developed a keyword- and subject heading–based search strategy and searched PubMed, EBSCOhost, Scopus, and Web of Science for studies on discharge readiness and postpartum discharge. The search was re-run on 4 October 2026 using the same time frame and eligibility criteria; only one new study was identified. Citation tracking and reference list screening of included studies were used to identify additional relevant sources, including non-indexed studies, that may not have surfaced in the database searches.

All retrieved studies were imported into Covidence, a web-based platform for managing large volumes of data in evidence synthesis [24,25]. First, titles and abstracts were screened to identify potentially relevant studies. Second, full texts were assessed against predefined inclusion and exclusion criteria (Table 2). This structured approach ensured rigorous, transparent selection of evidence relevant to the review objectives.

- Search Strategy

The search strategy was designed to comprehensively and systematically identify relevant literature in line with the review’s objectives and inclusion criteria. Following Arksey and O’Malley’s framework and PRISMA-ScR guidelines, a broad, sensitive search was conducted in PubMed, EBSCOhost, Scopus, and Web of Science. For each database, tailored combinations of Medical Subject Headings (MeSH) and free-text terms were used, with Boolean operators (e.g., AND, OR), truncation (e.g., “postpartum*” to capture variants such as “post-partum”), and filters applied as needed to refine results.

Postpartum discharge OR Postpartum preparedness AND Maternal readiness.

Postpartum AND Mothers OR Women AND Health facility AND Discharge readiness (Supplementary File S2: Search strategy).

In addition to peer-reviewed articles, grey literature, including dissertations, was searched to identify studies that might have been missed; none were identified. All retrieved citations were imported into COVIDENCE.

- Eligibility

Studies meeting the predefined inclusion criteria were screened by title and abstract [21]. Eligible studies focused on predominantly low-risk postpartum women who delivered in health facilities. Inclusion and exclusion criteria were entered into Covidence to support screening; Covidence served as a management tool, not an automated exclusion system. All reviewers conducted the literature search independently. Two reviewers independently screened titles/abstracts and full texts against eligibility criteria, with a third reviewer co-checking. Discrepancies were assessed against the eligibility criteria to reach inclusion consensus.

#### 2.1.4. Charting the Data and Data Extraction

Two reviewers independently extracted and charted the data, with a third reviewer verifying extraction. Disagreements were resolved using the inclusion criteria, and only eligible studies were synthesized. A predeveloped extraction table was used to record essential details under predetermined headings: author, year, title, research topic, country, study population/participants, sample size, study design, mode of birth, and findings relevant to the SCR. Guided by Arksey and O’Malley’s framework, this systematic process supported accuracy, completeness, and reliability.

#### 2.1.5. Organizing and Categorizing Key Findings

Data analysis used both deductive and inductive content analysis [26,27] aligned with Arksey and O’Malley’s iterative framework [22]. Data from six included studies was analyzed using a deductive-inductive content analysis approach. The first author performed the initial coding. For the deductive element, coding was directed by pre-established categories based on the review aims and key concepts drawn from the literature; the inductive element enabled new codes to be generated. The second, third, and fourth authors then independently co-checked the codes, and any disagreements were discussed until consensus was achieved.

Similar codes were compared and grouped into categories to develop eight themes. The themes were checked against the codes and extracted data to ensure they reflected evidence from the included studies. Coding, categorization, and theme development proceeded iteratively, with adjustments made as needed through discussion and comparison with the extracted findings.

The eight themes were education (three studies), confidence (two studies), care skills (three studies), information gaps (two studies), woman-centred care (two studies), maternal health (one study), early discharge (one study), and health system factors (four studies). Single studies could inform more than one theme. (Table 3).

- Summarizing and reporting results

Following Arksey and O’Malley’s fifth step, results were systematically analyzed to identify patterns and synthesize findings into clear, concise insights. The discussion situated the findings in relation to existing literature and then moved towards recommendations for practice and further investigation. The results were structured and aligned with the review’s objectives, ensuring that the key findings were meaningful and relevant to the review question.

- Rigor

**Figure 1:**
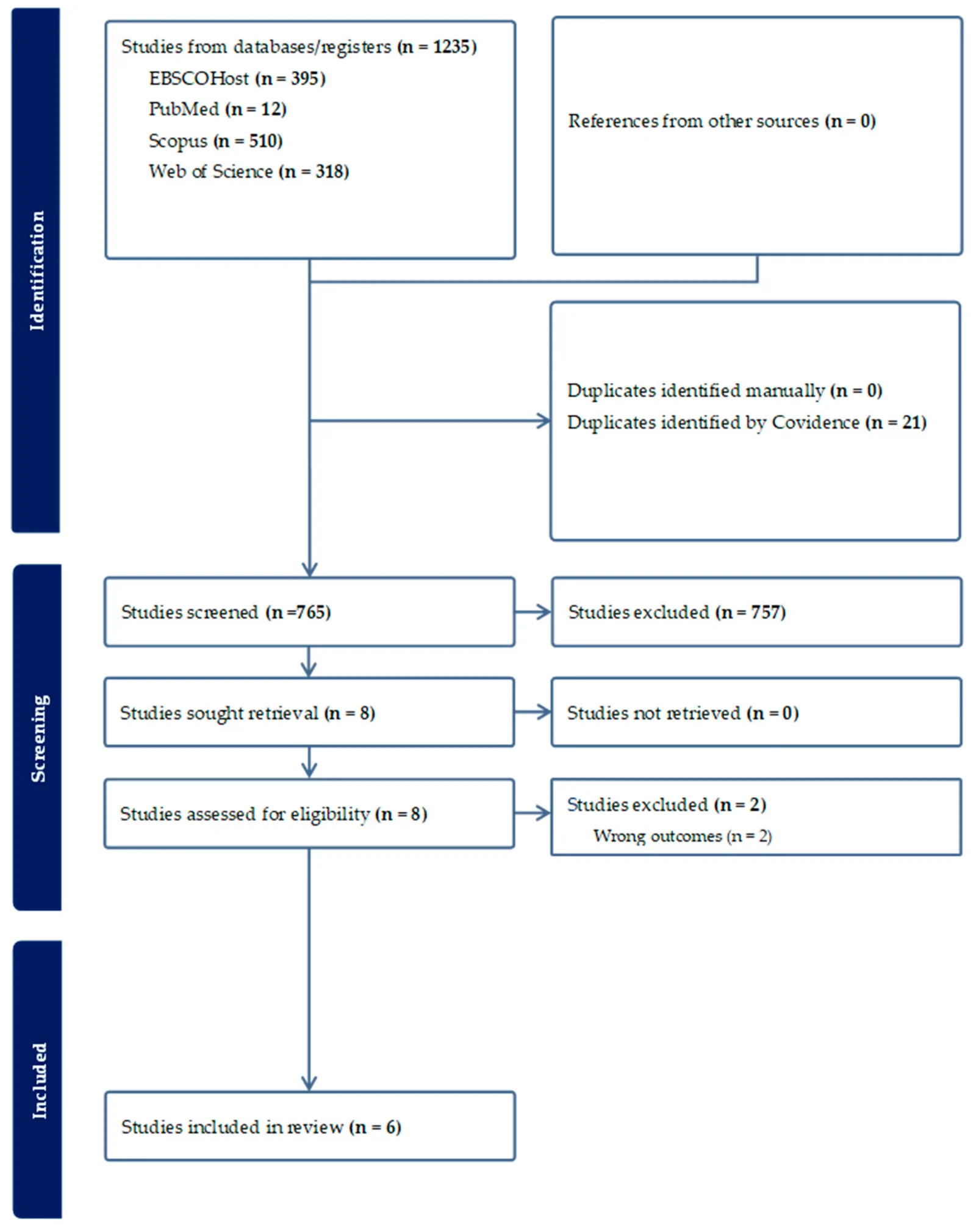
PRISMA flow chart illustrates the review process.

Ensuring rigor is essential to high research standards, as it reduces bias and strengthens the credibility of findings. The research protocol was registered with the Open Science Framework (OSF) https://osf.io/khmq2/overview (11 September 2026) to promote transparency. A systematic, transparent approach to formulating the research question, defining inclusion/exclusion criteria, and synthesizing data reinforces the review’s rigor. The review process is documented in the PRISMA flow diagram (Figure 1) and PRISMA-ScR checklist. Consensus among the three reviewers and careful reporting of primary study findings as originally presented minimized oversights and strengthened the review’s validity [28].

## 3. Results

### 3.1. Characteristics of Studies Included

This review synthesized evidence from six studies on discharge preparation and support for postpartum mothers, focusing on readiness for discharge and transition to home-based care across diverse global maternity settings. Collectively, the studies included more than 15,000 women, although the sample sizes and study designs varied substantially. The settings included both high-income and low- and middle-income countries. In Africa, a cross-sectional descriptive survey in Ghana assessed women’s understanding of postpartum care, practices, barriers, and educational needs [29], while a multi-country cross-sectional analytical study across four African nations (Burkina Faso, Ghana, Kenya, and Tanzania) identified factors associated with early discharge after vaginal birth [30]. In Asia, two quasi-experimental pre-post intervention studies were conducted, one in India [31] and one in Turkey [32]. In Australia, a mixed-methods study in New South Wales investigated postnatal care from childbirth to discharge within maternity care services [33]. The narrative synthesis is provided in the next section (Table 4).

#### Narrative Synthesis

Only six studies were eligible, indicating a gap in studies focusing on discharge education and preparation. The studies included conceptualized and assessed postpartum discharge readiness in varied ways, with no shared operational definition. Instead, readiness was reflected through different aspects of women’s preparation for discharge and transition home. One study described postpartum care needs in relation to women’s understanding of care, practices, barriers, and educational needs [29]. Another examined early discharge after vaginal delivery and factors influencing discharge timing, highlighting circumstances around the facility-to-home transition [30]. A further study focused on mothers’ knowledge, attitudes, and practices regarding neonatal care and preparedness to care for the newborn after discharge [31]. One study assessed mothers’ discharge readiness and self-confidence following postpartum education [32]. Another examined the postnatal care journey from birth to discharge, including care experiences and preparation during the transition [33]. One study compared single room and traditional maternity care using validated questionnaires, measuring mothers’ perceived readiness for hospital discharge alongside satisfaction and length of stay, and thereby framing readiness as an outcome of the care environment and continuity of providers [34]. Overall, postpartum discharge readiness comprises related dimensions: knowledge and education, maternal self-confidence, preparedness for newborn care, understanding of postpartum self-care, recognition of care needs, the care environment and continuity of care, and transition to home, though studies emphasized these differently. Accordingly, this review mapped how the literature described and assessed these components rather than applying a single predefined definition. Table 5 shows how the concept varies across studies.

The narrative synthesis identified eight interrelated themes influencing postpartum discharge readiness among mothers following childbirth. These themes are: (1) postpartum education as a foundation for discharge readiness, (2) maternal self-confidence and empowerment for self-care and infant care, (3) acquisition of maternal and newborn care knowledge and practical skills, (4) information gaps and unmet educational needs during the postnatal period, (5) woman-centred care and supportive healthcare relationships, (6) inadequate attention to maternal physical and mental health needs, (7) early discharge practices and their implications for preparation and support, and (8) health system and organizational factors influencing discharge preparation and transition. Together, these themes provide a comprehensive understanding of the individual, interpersonal, and health system factors that shape mothers’ readiness for discharge and their successful transition from hospital to home following childbirth. From the six studies, eight interconnected themes shaped mothers’ readiness for discharge after childbirth. (Table 6).

The review highlighted that postpartum education was associated with discharge readiness and maternal confidence [32], and that use of a structured checklist was associated with higher maternal knowledge [31]. Mothers who received single room maternity care also reported higher readiness for discharge and greater satisfaction than those receiving traditional care [34]. However, persistent gaps and inconsistencies were reported, with many women still describing unmet learning needs [29]. Overall, discharge readiness appears to be a complex, multidimensional process that is linked to high-quality education, psychosocial support, woman-centred care, the care environment, continuity of providers, and strong health system support for a safe transition home. Table 7 presents the themes identified across the six studies, along with explanations of how each is relevant to the purpose of this review.

## 4. Discussion

This scoping review identified postpartum education and discharge preparation as recurring components of postpartum discharge readiness across the included studies [29,30,31,32,33,34], while one study also suggested that the model of care and the care environment may influence readiness [34]. However, the evidence base was limited and heterogeneous, and the relative contribution of individual components to discharge readiness could not be established.

### 4.1. Components of Readiness

#### 4.1.1. Maternal Confidence

Maternal confidence emerged as an important aspect of discharge readiness. It may be supported through information, hands-on demonstrations, counselling, and supportive interactions with healthcare professionals [31,32]. Well-prepared mothers may feel better able to care for themselves and their newborns at home. This confidence may be particularly important during the transition to home-based care, where mothers are required to apply acquired knowledge and skills, make decisions, and seek help when needed. However, the findings suggest that confidence represents only one dimension of discharge readiness. Readiness also encompasses maternal knowledge and practical caregiving skills [31,32,34], the quality of communication and support provided by healthcare professionals [33,34], and organizational conditions surrounding discharge [30,31,34]. Discharge preparation should therefore go beyond providing information to include opportunities to develop practical competence, confidence, and the ability to manage postpartum and newborn care after leaving the facility.

#### 4.1.2. Maternal Knowledge and Practical Skills

Another recurring aspect of discharge readiness was the development of maternal knowledge and practical skills. The included studies indicate that preparation for discharge involves both understanding relevant information and being able to apply this knowledge in postpartum and newborn care [31,32,34]. Key areas included breastfeeding, keeping the baby warm, umbilical cord care, immunization schedules, maternal health, and recognizing warning signs for illnesses [31,32]. Deficits in mothers’ awareness of postpartum danger signs suggest that discharge preparation may not adequately address key maternal health needs [29]. This contrast between gains following structured education and persistent information gaps suggests that the content, consistency, and practical orientation of discharge education warrant further attention. Rather than focusing solely on the provision of information, discharge preparation may benefit from ensuring that mothers have opportunities to develop and demonstrate the knowledge and practical skills needed for care at home.

#### 4.1.3. Gaps in Information Provided

Although the value of postpartum education is widely recognized, the included studies also identified gaps in the information provided to women before discharge. Women often reported insufficient guidance on physical recovery, infant care, emotional well-being, and warning signs requiring medical attention. Some women felt poorly informed before discharge [33], while limited knowledge of postpartum danger signs and expectations about recovery was also reported [29]. These findings suggest that the presence of discharge education does not necessarily ensure that women feel adequately prepared for the transition home. The nature, content, consistency, and practical relevance of the information provided may therefore be important considerations in supporting discharge readiness. Addressing information gaps may help women feel more confident in managing postpartum and newborn care and in recognizing situations requiring further professional support.

#### 4.1.4. Woman-Centred Care and Supportive Relationships

The findings also highlight the importance of woman-centered care and supportive healthcare relationships in the transition from facility-based to home-based care. In the included studies, women who received respectful communication, tailored support, emotional reassurance, and consistent follow-up reported more confidence about going home. Supportive professionals and continuity of care were associated with more positive postnatal experiences, while fragmented care and poor communication reduced satisfaction [33]. Similarly, mothers who received single room maternity care, with a consistent team of providers and no transfer between units, reported higher readiness for discharge than those receiving traditional maternity care [34]. However, because this comparison was cross-sectional and involved one hospital per model, the contribution of provider continuity itself cannot be isolated. Overall, these findings show that discharge readiness goes beyond clinical information to include emotional support, opportunities to ask questions, and acknowledgement of mothers’ individual needs and concerns.

#### 4.1.5. Health System and Organizational Factors

Health system and organizational factors also appear to shape discharge readiness. In one comparison of two hospitals, mothers receiving single room maternity care, in which labour, birth, and postpartum care take place in one room with the same team, differed in readiness for discharge from those receiving traditional care [34]. These findings suggest that how and where postnatal care is organized may influence readiness alongside the education mothers receive. Together with evidence that facility type and access influenced discharge practices [30] and structured checklists improved the consistency of educational delivery [31], they indicate that readiness depends on both individual preparation and the systems in which care is delivered. Further research should examine whether these associations hold across settings and care models.

#### 4.1.6. Baby-Centred Focus of Postnatal Care

Another key finding from one study is that postnatal care tends to prioritize infant health while giving limited attention to the mother’s recovery and well-being, as postpartum care was largely baby-centred, with many mothers reporting inadequate support for their physical recovery, mental health, and emotional needs [29]. This imbalance may be associated with lower discharge readiness, as maternal health is associated with caregiving capacity and overall family well-being. These findings suggest the need for a more holistic approach to discharge preparation that considers the health and well-being of both mother and newborn, rather than focusing predominantly on infant care.

#### 4.1.7. Early Discharge

The trend toward early postpartum discharge highlights the importance of ensuring adequate preparation before women leave the health facility. In a study conducted across four African countries, nearly one-third of women were discharged early after vaginal delivery [30]. Shorter postnatal stays may reduce the time for education, assessment, counselling, and supportive care, potentially limiting opportunities to address mothers’ preparation needs before discharge. This finding highlights the importance of well-structured discharge processes that ensure essential information, practical skills, and opportunities for questions are addressed within the time available. Such processes may be particularly important in settings where early discharge is common, and continuity of postnatal support after leaving the facility is limited.

### 4.2. Interventions to Improve Discharge Readiness

#### 4.2.1. Postpartum Education and Structured Discharge Preparation

Postpartum education was also identified as an important component of discharge preparation. Organized health education on postpartum recovery, newborn care, breastfeeding, hygiene, warning signs, and self-care strengthens mothers’ readiness for discharge. Checklist-guided programs were also used to structure educational delivery [31]. Together, these findings suggest discharge preparation is more than information transfer. Its potential contribution to readiness appears to involve both acquisition of practical knowledge and development of maternal confidence to use this knowledge in home care.

Health services should consider models of care and the care environment, such as single-room maternity care, to support readiness and satisfaction [34].

#### 4.2.2. Associated Outcomes

Outcomes of postpartum education and discharge preparation fell into four areas. Structured educational interventions improved maternal knowledge and practice, and a checklist-guided program greatly increased maternal knowledge [31,32]. Second, postpartum teaching improved mothers’ discharge preparedness and self-confidence [32], and education and counselling were reported to improve maternal self-efficacy and caregiving confidence [31,32]. Together, these findings suggest that practical preparation may contribute to maternal competence.

Third, satisfaction with postnatal care was higher among women who received respectful communication, tailored support, emotional reassurance, and consistent follow-up [33]. In comparison of care models, mothers who received single room maternity care reported significantly higher satisfaction and readiness for discharge than those receiving traditional maternity care [34].

Fourth, length of stay differed by care model. Single room maternity care was associated with shorter newborn length of stay, while mother length of stay did not differ significantly after adjusting for other variables [34].

These outcomes should be interpreted cautiously. Most of these studies used cross-sectional or non-randomized designs, small samples, self-reported measures, and short follow-up, so causal links cannot be inferred, and it is unclear whether gains in confidence, knowledge, or caregiving practices were maintained over time [31,32,34]. The care-model comparison involved only one hospital per model, so differences in readiness and satisfaction may partly reflect hospital-level factors rather than the care model alone [34].

## 5. Limitations

This review has limitations. The limited number of eligible studies highlights a gap in the available evidence on postpartum discharge readiness and preparation. Although the small evidence base restricts the breadth of the findings, it also demonstrates the need for further research into how discharge readiness is conceptualized, assessed, and addressed across different healthcare contexts. The findings should therefore be interpreted in relation to the limited available literature rather than as a comprehensive representation of all dimensions of postpartum discharge readiness. However, a scoping review is also valuable for identifying where evidence is scarce. Only six studies met inclusion criteria, yielding limited evidence on postpartum discharge readiness and preparation. Heterogeneity in designs, concepts, terminology, and outcomes hindered comparison and obscured patterns. Differences in maternity and healthcare contexts may limit transferability. As a scoping review, it mapped evidence rather than assessing intervention effectiveness or causality. Still, it summarizes current evidence and identifies gaps in postpartum discharge knowledge.

The findings underscore the need for structured, individualized postnatal education and discharge preparation covering maternal and newborn care, danger signs, breastfeeding, self-care, and follow-up. Providers should confirm women’s readiness and understanding before discharge and ensure continuity of care afterwards, and health services should consider how models of care and the care environment, such as single room maternity care, support readiness and satisfaction. Policy should support standardised guidelines, adequate staffing and resources, and quality monitoring of postnatal care. Future research should use larger multicenter longitudinal studies to assess long-term effects on maternal and neonatal outcomes and the implementation costs and provider outcomes of different care models, and explore women’s experiences, unmet needs, and barriers to postnatal care.

## 6. Conclusions

Readiness for postpartum discharge is multidimensional. It is shaped by education, maternal confidence, knowledge and practical skills, woman-centred care, attention to the mother’s well-being, discharge timing, the care environment, and continuity of providers, as well as by health system factors such as staffing levels, organizational structures, discharge protocols, and the availability of educational resources. Strengthening these processes and standardizing discharge procedures may improve the quality and consistency of discharge preparation, which calls for commitment from HCPs and administrators.

It is recommended that structured, individualized discharge education covering maternal and newborn care, danger signs, breastfeeding, and follow-up be provided. HCPs should confirm women’s understanding before discharge and ensure continuity of care afterwards. However, the evidence base was limited to six heterogeneous studies, and approaches to defining and assessing discharge readiness were inconsistent. Further research should develop a clearer conceptual and measurement framework for discharge readiness, use larger multicenter longitudinal studies to assess long-term outcomes and the costs of different care models, and explore women’s unmet needs and barriers to postnatal care.

## Figures and Tables

**Table 1:** Population, Concept, and Context.

Population:	Postpartum women/mothers who gave birth in a healthcare facility and were preparing for or transitioning from maternity facility care to home.
Concept:	Postpartum discharge readiness is defined as the mother’s preparedness for discharge from facility-based maternity care, together with discharge preparation processes directed at the mother (assessment, education, planning, and follow-up arrangements) and the components that shape her transition from facility-based care to home.
Context:	Healthcare facilities and maternity care in global maternity care settings?

**Table 2:** Inclusion criteria and exclusion criteria.

Inclusion	Rationale
Literature published between 2014 and 2025	Selecting literature from January 2014 to December 2025 ensures that the evidence reflects current practices, policies, and standards of care in postpartum discharge.
Language	Studies published in English
Quantitative, qualitative, mixed-methods studies	Quantitative studies provide measurable outcomes and statistical insights, qualitative studies offer in-depth perspectives and experiences, and mixed-methods studies bridge both, enhancing the validity and richness of findings.
Studies focusing on low-risk postpartum women who gave birth in a health facility	To avoid confounding factors associated with high-risk pregnancies or home births that may skew perceptions or outcomes of readiness for discharge.
Studies focusing on postpartum readiness for discharge from a health facility	Excluding studies that focus on broader postpartum care without specifically addressing discharge preparedness, ensuring relevance to the review question.
Research centered on women in the early postpartum period (up to six weeks)	This timeframe ensures relevance to early postpartum health outcomes and interventions, capturing data that directly reflects the initial recovery and adjustment phase after delivery.

**Table 3:** Thematic coding and theme development process.

Six included studies One reviewer extracted findings; two reviewers independently verified			
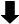			
Initial coding First author; deductive and inductive coding			
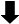			
Independent review of codes Second, third and fourth authors; discussion and consensus			
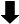			
Category development Related codes compared and grouped into categories			
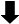			
Eight themes Checked against codes and extracted findings			
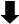			
Theme development and verification Eight themes reviewed against the codes and extracted findings			
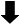			
Iterative review and refinement Codes, categories and themes revised as needed following discussion and comparison with the extracted findings			
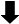			
Final themes			
Theme 1 (*n* = 3) Education	Theme 2 (*n* = 2) Confidence	Theme 3 (*n* = 3) Care skills	Theme 4 (*n* = 2) Information gaps
Theme 5 (*n* = 2) Woman-centred	Theme 6 (*n* = 1) Maternal health	Theme 7 (*n* = 1) Early discharge	Theme 8 (*n* = 4) Health system

**Table 4:** Data Extraction Characteristics of included studies.

Author/s & Year	Study Setting	Aims/Objectives	Study Design	Sample/Population	Data Collection & Analysis	Main Results (Relevant to Review Question)
Adams et al. 2023. [29]	The study was conducted in four health centers (Kanvilli, Choggu, Kalpohin, and Bagabaga) in the Sagnarigu District in Tamale, Ghana. Participants were postpartum women receiving postnatal care in these facilities.	To gather information on women’s knowledge of postpartum care and complications- applications, recovery practices after childbirth, perceived barriers to receiving care during the postpartum period, and educational needs in four health centers.	A descriptive qualitative study design was used. Data were collected through focus group discussions (FGDs).	54 postpartum women aged 19–44 years who had delivered within the previous year. Participants were divided into women less than 6 months postpartum and women 6–12 months postpartum.	Eight focus groups were conducted, with 6–8 women in each group. Discussions were audio-recorded, transcribed, translated into English when necessary, and analyzed using thematic analysis.	Six main themes emerged from the study: (1) baby-focused postpartum care, (2) postpartum practices, (3) inadequate knowledge of postpartum danger signs, (4) barriers to accessing postpartum care, (5) experiences of poor mental health, and (6) the need for postpartum education. The study highlighted that postpartum care focused mainly on the baby, while mothers’ physical and mental health needs were often neglected
Tekeba et al. 2024 [30]	Four African countries: Burkina Faso, Ghana, Kenya, and Tanzania	The study aimed to assess the prevalence of early discharge among women who had vaginal delivery in health facilities.	cross-sectional	14,942 women who gave birth at a health facility	The data used were obtained from the Demographic Health Survey of four African countries between 2021 and 2023. They used pretested and validated questionnaires to collect data. A multilevel mixed-effect binary logistic regression model was used to analyse the data	The overall prevalence of early discharge following health facility vaginal delivery in four African countries was 30.91%. Overall, first-born babies, women with a high maternal body mass index, women with multiple pregnancies, women who reside in Burkina Faso, and women who reside in Ghana had lower odds of early discharge following health facility vaginal delivery. On the other hand, women having no difficulty accessing health facilities and women delivering in private health facilities had higher odds of early discharge following health facility vaginal delivery.
Arunakumar et al., 2022. [31]	The study was conducted in the postnatal wards of K.C. General Hospital, Department of Health and Family Welfare Services, Bengaluru, Karnataka, India.	To improve newborn and maternal self-care practices among postnatal mothers through quality improvement techniques and a checklist intervention. Another objective was to increase awareness regarding newborn and perinatal care practices included in the Mother and Child Protection (MCP) Card.	This was a 4-month hospital-based prospective interventional study conducted using four Plan-Do-Study-Act (PDSA) cycles	A total of 102 postnatal mothers were enrolled in the study, and 78 mothers completed follow-up. Participants were mothers admitted to the postnatal wards. Mothers whose newborns required intensive neonatal care were excluded.	Baseline maternal knowledge was assessed using a structured checklist questionnaire covering 15 neonatal and maternal self-care parameters. Mothers received counselling and education using the MCP Card, posters, flip charts, and demonstrations. A follow-up assessment was performed after 5–7 days. Statistical analysis included the Wilcoxon signed-rank test and linear regression.	The checklist intervention significantly improved mothers’ knowledge, attitude, and practices regarding neonatal and self-care practices in all four PDSA cycles (*p* < 0.05). Median checklist scores increased substantially after counselling. Awareness and use of the MCP Card improved from less than 5% to 38%. Mothers demonstrated better understanding of breastfeeding, keeping the baby warm, umbilical cord care, vaccination, danger signs, and maternal self-care.
Sönmez & Mamuk. 2021. [32]	Hospital maternity setting (Turkey)	To determine the effect of postpartum education on primiparous mothers’ readiness for hospital discharge and maternal self-confidence	Quasi-experimental study	102 primiparous postpartum mothers (50 patients for the experimental group and 52 for the control group).	Maternal self-confidence scale; statistical analysis	Women reported that postpartum education significantly improved readiness for discharge and increased self-confidence. The education also prepared the new mothers with the ability to care for themselves and their newborns after discharge
Schmied et al., 2024. [33]	Maternity service in New South Wales, Australia	To explore women’s experiences of postnatal care from birth to hospital discharge	Mixed-methods study	15 postnatal women Postpartum period: 10–14 days	Survey and in-depth interviews; thematic analysis for qualitative data and descriptive statistics for quantitative data	Women described a range of experiences with postnatal care, with some reporting a lack of continuity, limited information, and insufficient emotional support. Positive experiences were often linked to supportive healthcare staff and consistent care throughout the postnatal period. These findings suggest that postnatal services could be improved by placing greater focus on woman-centred care.
Hall et al. 2023 [34]	Two large tertiary hospitals in Western Canada, one providing single room maternity care and the other traditional maternity care, with the same nurse staffing ratios.	To compare the two care models on mothers’ readiness for discharge, satisfaction, and length of stay, while accounting for other factors that could influence the results.	Cross-sectional comparison of mothers in the two hospitals.	Postpartum mothers from both hospitals, with more participants from the single room maternity care site. The two groups differed in language, birthplace, and income.	Mothers completed validated questionnaires on readiness for discharge in hospital and on satisfaction two weeks after discharge. Length of stay and clinical details came from health authority records. Group differences were tested with statistical models that adjusted for other variables.	Mothers receiving single room maternity care felt more ready for discharge and were more satisfied, particularly regarding knowledge of self-care and baby care, emotional readiness, and expected support at home. Prenatal classes also contributed to readiness and satisfaction. The authors recommend research on provider outcomes and implementation costs.

**Table 5:** Variation in concept across the six included studies.

Study	How Discharge Readiness/Preparation Was Represented	Relevant Components
Adams et al. [29]	Women’s understanding and preparation for postpartum care	Understanding of postpartum care; postpartum practices; barriers; educational needs
Tekeba et al. [30]	Transition from facility to home reflected through timing of discharge	Early discharge; factors associated with discharge timing; transition following vaginal birth
Arunakumar et al. [31]	Maternal preparedness for neonatal care	Knowledge; attitudes; practices; newborn care
Sönmez & Mamuk [32]	Explicit assessment of readiness for hospital discharge	Discharge readiness; postpartum education; maternal self-confidence
Schmied et al. [33]	Postnatal journey and preparation from birth to discharge	Postnatal care experiences; care received; transition to discharge
Hall et al. [34]	Explicit assessment of perceived readiness for hospital discharge as an outcome of the care model (single room versus traditional maternity care)	Discharge readiness; maternal satisfaction; continuity of providers and care environment

**Table 6:** Eight themes identified across the six studies.

Factors Influencing Postpartum Discharge Readiness						
Postpartum education as a foundation for discharge readiness	Maternal self-confidence and empowerment for self-care and infant care	Acquisition of maternal and newborn care knowledge and practical skills	Information gaps and unmet educational needs during the postnatal period	Woman-centred care and supportive healthcare relationships	Inadequate attention to maternal physical and mental health needs	Health system and organizational factors influencing discharge preparation and transition

**Table 7:** Themes and descriptions.

Theme	Description of Theme	Evidence from Studies	Relevance to Discharge Readiness for Postpartum Mothers
1. Postpartum education as a foundation for discharge readiness	Structured postpartum education prepares mothers with essential knowledge and skills before discharge, focusing on maternal recovery, newborn care, breastfeeding, danger signs, hygiene, and self-care.	Postpartum education significantly improved mothers’ readiness for discharge and self-confidence, with mothers feeling better prepared for self and newborn care at home [32]. A substantial unmet need for postpartum education existed [29]. Maternal knowledge improved significantly after the checklist-based intervention [31].	Education is a central component of discharge preparation and is associated with mothers’ postpartum skills for self and newborn care.
2. Maternal self-confidence and empowerment for self-care and infant care	Discharge preparation interventions build mothers’ confidence in caring for themselves and their babies through information, practical demonstrations, counselling, and supportive interactions with healthcare providers.	Increased maternal self-confidence after postpartum education was reported [32]. Mothers felt more capable of managing maternal and newborn care needs at home, and counselling and checklist interventions improved mothers’ attitudes and confidence in neonatal and maternal care practices [31].	Maternal confidence is associated with readiness for discharge. Mothers who feel assured and well-informed tend to report better adjustment to caregiving and a greater likelihood of seeking assistance when required.
3. Acquisition of maternal and newborn care knowledge and practical skills	After discharge, mothers need both theoretical knowledge and practical skills for safe care, including breastfeeding, thermal and cord care, immunization, maternal recovery, hygiene, and recognizing warning signs.	Improved knowledge and practices related to breastfeeding, keeping the baby warm, umbilical cord care, vaccination schedules, maternal self-care, and recognition of neonatal danger signs were reported [31]. In comparison, some mothers felt more competent in caring for themselves and infants following education [32]. In contrast, there was inadequate knowledge of postpartum danger signs among many women [29].	Knowledge and skills acquisition are associated with discharge readiness, and with mothers’ reported ability to manage common postpartum and newborn care needs without constant professional supervision.
4. Information gaps and unmet educational needs during the postnatal period	Despite available postnatal services, many mothers reported inadequate information on postpartum recovery, newborn care, emotional well-being, and warning signs needing medical attention.	Women frequently reported receiving limited information before discharge [33]. Mothers lacked knowledge regarding postpartum danger signs and expressed a need for additional education. Mothers reported uncertainty about what to expect during recovery and when to seek healthcare [29].	Information gaps are associated with lower discharge readiness, and with reports of anxiety, reduced confidence, delayed help-seeking behaviour, and difficulties managing health concerns after discharge.
5. Woman-centred care and supportive healthcare relationships	Positive discharge experiences are linked to individualized care, respectful communication, emotional support, and continuity of care. Woman-centred approaches recognize mothers’ unique needs, preferences, and concerns in the postnatal period.	Women who experienced supportive healthcare staff and continuity of care reported more positive postnatal experiences. Conversely, fragmented care and poor communication reduced satisfaction with postnatal services [33]. Mothers who received single-room maternity care with the same care team throughout labour, birth, and postpartum reported significantly higher satisfaction than those receiving traditional maternity care with changing providers [34].	Woman-centred care is associated with higher discharge readiness. Features include individualized support, encouragement to ask questions, and ongoing emotional support during the transition home. Continuity of providers is also associated with greater readiness and satisfaction during the transition home.
6. Inadequate attention to maternal physical and mental health needs	Postnatal care often prioritizes infant health while overlooking mothers’ physical recovery, emotional well-being, and mental health, leaving a gap in comprehensive discharge preparation.	Postpartum care was largely baby-focused. Mothers reported experiences of poor mental health and inadequate attention to their own recovery needs. The study highlighted insufficient education regarding maternal health concerns and emotional well-being [29].	Readiness for discharge is associated with attention to both maternal and newborn health. Where maternal physical or psychological needs are overlooked, studies suggest this may be linked to poorer recovery, reduced caregiving capacity, and lower post-discharge well-being.
7. Early discharge practices and their implications for preparation and support	Early discharge shortens the time available for education, counselling, assessment, and support before mothers go home, increasing the need for efficient discharge preparation.	Women across four African countries experienced early discharge following vaginal delivery. Factors such as facility type, accessibility of healthcare services, maternal characteristics, and country of residence influenced discharge timing [30].	Early discharge is associated with a need for mothers to receive adequate information, practical skills training, discharge planning, and follow-up before leaving the facility.
8. Health system and organizational factors influencing discharge preparation and transition	Discharge preparation depends on system-level factors, including staffing, continuity of care, service organization, models of care and the care environment, institutional policies, educational resources, and discharge standard operating procedures.	Inconsistencies in postnatal care, continuity of care, and information provision [33]. The type of health facility and access influenced discharge practices [30]. A structured checklist improved consistency in educational delivery [31]. Mothers receiving single room maternity care, with labour, birth, and postpartum care in one room with a consistent team of providers, reported significantly higher readiness for discharge and satisfaction than those receiving traditional maternity care [34].	Health-system factors are associated with whether mothers receive comprehensive, standardised, and supportive discharge preparation. Models of care that preserve continuity of providers and a consistent care environment are associated with higher readiness and satisfaction, while fragmented services are associated with mothers feeling underprepared.

## Data Availability

No new data were created or analyzed in this study. Data sharing does not apply to this article.

## Supplementary Materials

The following supporting information can be downloaded at: https://www.mdpi.com/article/10.3390/healthcare14213827/s1, Supplementary File S1: The review process; Supplementary File S2: Search strategies.

## Abbreviations

The following abbreviations are used in this manuscript:

PCC	Population, Concept, Context
PPH	Postpartum haemorrhage
WHO	World Health Organization

## References

[1] World Health Organization (2013). Postnatal Care for Mothers and Newborns: Highlights from the World Health Organization 2013 Guidelines.

[2] Leahy-Warren P., McCarthy G., Corcoran P. (2012). First-time mothers: Social support, maternal parental self-efficacy and postnatal depression. J. Clin. Nurs..

[3] Munabi-Babigumira S., Glenton C., Lewin S., Fretheim A., Nabudere H. (2017). Factors that influence the provision of intrapartum and postnatal care by skilled birth attendants in low- and middle-income countries: A qualitative evidence synthesis. Cochrane Database Syst. Rev..

[4] O’Hara M.W., McCabe J.E. (2013). Postpartum depression: Current status and future directions. Annu. Rev. Clin. Psychol..

[5] Dennis C.L. (2010). Postpartum depression peer support: Maternal perceptions from a randomized controlled trial. Int. J. Nurs. Stud..

[6] Yelland J., Sutherland G., Brown S.J. (2010). Postpartum anxiety, depression and social health: Findings from a population-based survey of Australian women. BMC Public Health.

[7] Carroli G., Mignini L. (2009). Episiotomy for vaginal birth. Cochrane Database Syst. Rev..

[8] Mousa H.A., Blum J., Abou El Senoun G., Shakur H., Alfirevic Z. (2014). Treatment for primary postpartum haemorrhage. Cochrane Database Syst. Rev..

[9] Sibai B.M. (2012). Etiology and management of postpartum hypertension-preeclampsia. Am. J. Obstet. Gynecol..

[10] Wallin N., Bergman L., Smith G.N. (2024). Pregnancy-associated cardiovascular risks and postpartum care: An opportunity for interventions aiming at health preservation and disease prevention. Best Pract. Res. Clin. Obstet. Gynaecol..

[11] Bouvier-Colle M.H., Pequignot F., Jougla E. (2001). Maternal mortality in France: Frequency, trends and causes. J. Gynecol. Obstet. Biol. Reprod..

[12] Say L., Chou D., Gemmill A., Tunçalp Ö., Moller A.B., Daniels J., Gülmezoglu A.M., Temmerman M., Alkema L. (2014). Global causes of maternal death: A WHO systematic analysis. Lancet Glob. Health.

[13] World Health Organization (2022). Trends in Maternal Mortality 2000 to 2020: Estimates by WHO, UNICEF, UNFPA, World Bank Group and UNDESA/Population Division.

[14] Khan K.S., Wojdyla D., Say L., Gülmezoglu A.M., Van Look P.F. (2006). WHO analysis of causes of maternal death: A systematic review. Lancet.

[15] Ochala E. (2023). Addressing postnatal challenges: Effective strategies for postnatal care. Contemporary Challenges in Postnatal Care.

[16] UNICEF (2023). Global Breastfeeding Scorecard 2023.

[17] Giannoni E., Agyeman P.K., Stocker M., Posfay-Barbe K.M., Heininger U., Spycher B.D., Bernhard-Stirnemann S., Niederer-Loher A., Kahlert C.R., Donas A. (2018). Neonatal sepsis of early onset, and hospital-acquired and community-acquired late onset: A prospective population-based cohort study. J. Pediatr..

[18] Koblinsky M., Moyer C.A., Calvert C., Campbell J., Campbell O.M.R., Feigl A.B., Graham W.J., Hatt L., Hodgins S., Matthews Z. (2016). Quality maternity care for every woman, everywhere: A call to action. Lancet.

[19] Marsh A. (2019). The importance of language in maternity services. Br. J. Midwifery.

[20] Pollock D., Peters M.D.J., Tricco A.C., Munn Z., Jia R.M., Alexander L., Pieper D., Evans C., Godfrey C.M., Brandão de Moraes E., Aromataris E., Lockwood C., Porritt K., Pilla B., Jordan Z. (2024). Scoping reviews (2026). JBI Manual for Evidence Synthesis.

[21] Tricco A.C., Lillie E., Zarin W., O’Brien K.K., Colquhoun H., Levac D., Moher D., Peters M.D.J., Horsley T., Weeks L. (2018). PRISMA extension for scoping reviews (PRISMA-ScR): Checklist and explanation. Ann. Intern. Med..

[22] Arksey H., O’Malley L. (2005). Scoping studies: Towards a methodological framework. Int. J. Soc. Res. Methodol..

[23] Sullivan I., DeHaven A., Mellor D. (2019). Open and Reproducible research on Open Science Framework. Curr. Protoc. Essent. Lab. Tech..

[24] Babineau J. (2014). Product review: Covidence (systematic review software). J. Can. Health Libr. Assoc..

[25] Harrison H., Griffin S.J., Kuhn I., Usher-Smith J.A. (2020). Software tools to support title and abstract screening for systematic reviews in healthcare: An evaluation. BMC Med. Res. Methodol..

[26] Kyngäs H. (2019). Inductive content analysis. The Application of Content Analysis in Nursing Science Research.

[27] Elo S., Kyngäs H. (2008). The qualitative content analysis process. J. Adv. Nurs..

[28] Aromataris E., Lockwood C., Porritt K., Pilla B., Jordan Z. (2024). JBI Manual for Evidence Synthesis.

[29] Adams Y.J., Miller M.L., Agbenyo J.S., Ehla E.E., Clinton G.A. (2023). Postpartum care needs assessment: Women’s understanding of postpartum care, practices, barriers, and educational needs. BMC Pregnancy Childbirth.

[30] Tekeba B., Dessie M.T., Tamir T.T., Zegeye A.F. (2024). Early discharge in health facilities following vaginal delivery and its associated factors among mothers in four African countries: A cross-sectional study. BMJ Open.

[31] Arunakumar S.P., Ramabhatta S., Lakshmipathy S.R., Raghunandan B.G., Rashmi K., Kumaraswamy D., Mol P.P., Anushree C. (2022). Enhancing the quality of neonatal care by improving mothers’ knowledge, attitude, and practice through checklist intervention. J. Clin. Neonatol..

[32] Sönmez B., Mamuk R. (2021). The effects of postpartum education in primipara mothers on their readiness for hospital discharge and maternal self-confidence. Med. J. Bakirkoy.

[33] Schmied V., Myors K., Burns E., Curry J., Pangas J., Dahlen H.G. (2024). A mixed-methods study of the postnatal care journey from birth to discharge in a maternity service in New South Wales, Australia. BMC Health Serv. Res..

[34] Hall M., Afzal A., White D.E. (2023). Single Room Maternity Care Versus Traditional Maternity Care: A Cross-Sectional Study Examining Differences in Mothers’ Perceptions of Readiness for Discharge and Satisfaction and Health Outcomes. Can. J. Nurs. Res..

