# Supplementary material for: Postpartum Discharge Readiness and Preparation: A Scoping Review
Source: Healthcare (Basel). 2026 Nov 9;14(21):3827. doi: 10.3390/healthcare14213827 (PMC13762888; doi:10.3390/healthcare14213827)
Supplement: Supplementary file 1 [file healthcare-14-03827-s001.zip › Supplementary File S1 Review process 14.10.2026.pdf]

Supplementary File S1: The Review Process

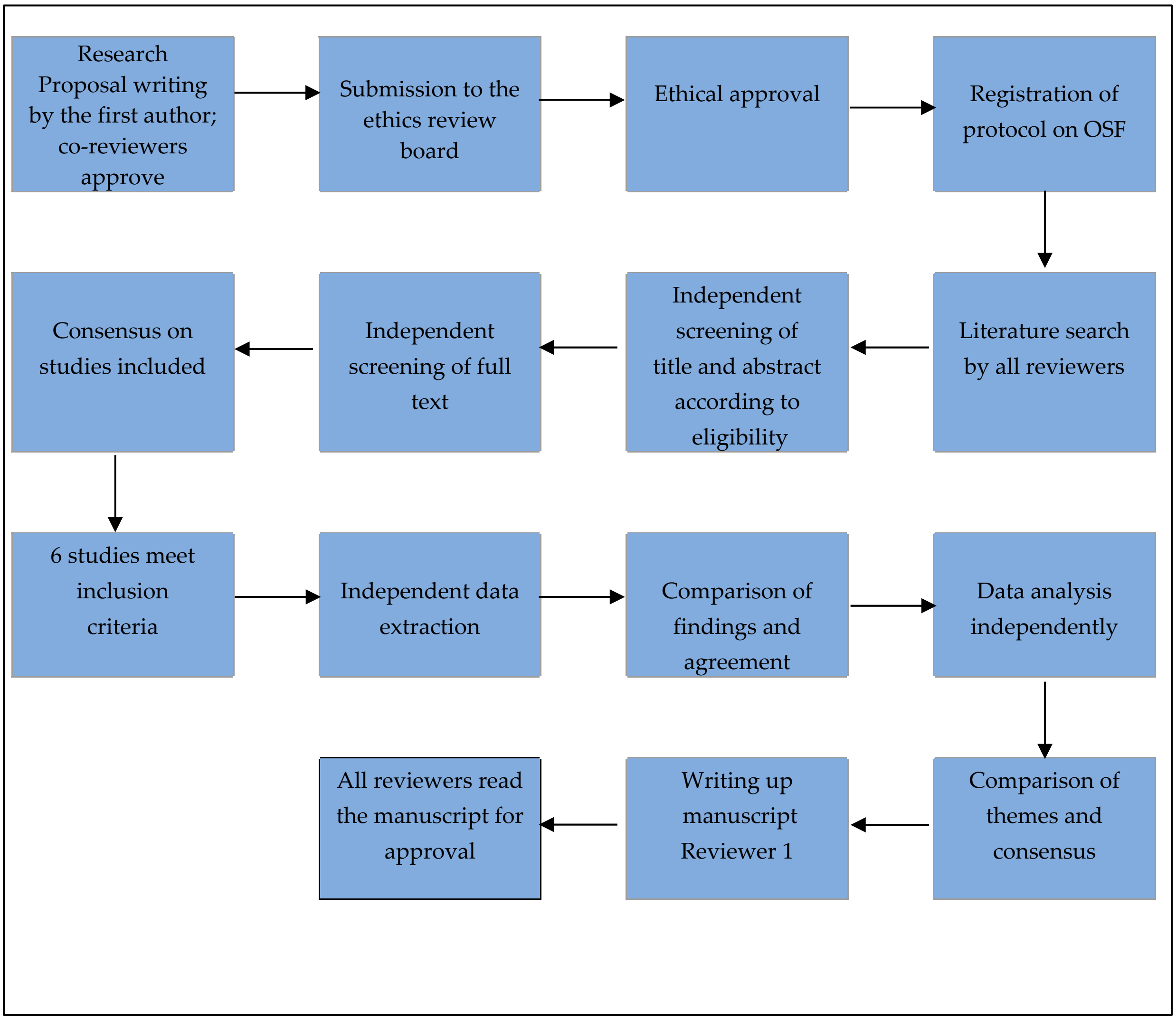

Figure S1: The steps undertaken in conducting the scoping review.
