# Supplementary material for: Postpartum Discharge Readiness and Preparation: A Scoping Review
Source: Healthcare (Basel). 2026 Nov 9;14(21):3827. doi: 10.3390/healthcare14213827 (PMC13762888; doi:10.3390/healthcare14213827)
Supplement: Supplementary file 1 [file healthcare-14-03827-s001.zip › Supplementary File S2 Search strategies 14.10.2026.pdf]

Supplementary File S2: Search strategies

Review on postpartum discharge readiness and preparation.

Database	Search strategy	Date searched	Limits applied
**PubMed**<br>12 HITS	(postpartum OR post-partum OR postnatal OR post-natal OR puerper* OR "new mother" OR "new mothers" OR "postpartum women" OR "postnatal women") AND ("discharge readiness" OR "readiness for discharge" OR "discharge preparation" OR "discharge preparedness" OR "discharge planning" OR "discharge teaching" OR "discharge education" OR "discharge instruction" OR "discharge instructions" OR "early discharge" OR "postpartum discharge" OR "postnatal discharge" OR "transition to home" OR "transition from hospital to home" OR "transitional care")	04102026	Year of publication: 2014-2025<br>Comparative study, evaluation study<br>Text availability: Abstract, Full Text<br>Type of research:<br>Evaluation study, Patient education, Interview, Personal Narrative, Introductory Journal article, Patient Education Handout<br>Adult, 19 years plus, Female
**Scopus**<br>**510 HITS**	(postpartum OR "post-partum" OR postnatal OR "post-natal" OR puerper* OR mother* OR maternal) AND ("discharge readiness" OR "readiness for discharge" OR "discharge prepar*" OR "discharge planning" OR "discharge teaching" OR "discharge education" OR "discharge instruction*" OR "early discharge" OR "postpartum discharge" OR "postnatal discharge" OR "hospital discharge" OR "transition to home" OR "transition from hospital to home" OR "care transition*" OR "transitional care" OR "continuity of care") AND (hospital* OR "health care facilit*" OR "healthcare facilit*" OR "maternity ward*" OR "maternity unit*" OR "maternity care")	04102026	Limited to Nursing, Articles, English Language<br><br>2014-2025<br><br>Nursing, Mothers, Mother, Postnatal period, Maternal Care, Education, Postpartum, Midwife, Puerperium, Postnatal care, Hospital Discharge, Patient Discharge
**Web of Science**<br>**318 Hits**	(postpartum OR post-partum OR postnatal OR post-natal OR puerper* OR mother* OR maternal)<br>AND<br>("discharge readiness" OR "readiness for discharge" OR "discharge prepar*" OR "discharge planning" OR "discharge teaching" OR "discharge education" OR "discharge instruction*" OR "early discharge" OR "postpartum discharge" OR "postnatal discharge" OR "hospital discharge" OR "transition to home" OR "transition from hospital to home" OR "care transition*" OR "transitional care" OR "continuity of care")<br>AND	04102026	Open Access and Article (Document Types) and Obstetrics Gynecology or Health Care Sciences Services (Research Areas) and JOURNAL OF OBSTETRICS AND GYNAECOLOGY CANADA or JOURNAL OF MIDWIFERY WOMENS HEALTH or JOURNAL OF OBSTETRIC GYNECOLOGIC NEONATAL NURSING or MIDWIFERY or INTERNATIONAL BREASTFEEDING JOURNAL or REPRODUCTIVE HEALTH or BMC HEALTH SERVICES RESEARCH or MATERNAL AND CHILD HEALTH JOURNAL or NURSING OPEN or BIRTH ISSUES IN PERINATAL CARE or EUROPEAN JOURNAL OF MIDWIFERY or HEALTH SA GESONDHEID or JOURNAL OF ADVANCED NURSING or SEXUAL REPRODUCTIVE HEALTHCARE OFFICIAL JOURNAL OF THE SWEDISH ASSOCIATION OF MIDWIVES or FRONTIERS IN GLOBAL WOMENS HEALTH or BMC WOMENS HEALTH or WOMENS HEALTH ISSUES or

Database	Search strategy	Date searched	Limits applied
	(hospital* OR "health care facilit*" OR "healthcare facilit*" OR "health facilit*" OR "maternity ward*" OR "maternity unit*" OR "maternity care")		WOMEN AND BIRTH JOURNAL OF THE AUSTRALIAN COLLEGE OF MIDWIVES or WOMEN AND BIRTH or BMC PREGNANCY AND CHILDBIRTH (Publication/Source Titles) and Article (Document Types) and Web of Science Core Collection (Database) and Open Access and 2025 or 2024 or 2023 or 2022 or 2021 or 2020 or 2019 or 2018 or 2017 or 2016 or 2015 or 2014 (Publication Years)
**EBSCOhost (Academic Search Complete)**<br><br>**395**	postpartum OR post-partum OR postnatal OR post-natal OR puerper* OR mother* OR maternal<br>"Discharge readiness" OR "readiness for discharge" OR "discharge prepar*" OR "discharge planning" OR "discharge teaching" OR "discharge education" OR "discharge instruction*" OR "early discharge" OR "postpartum discharge" OR "postnatal discharge" OR "hospital discharge" OR "transition to home" OR "transition from hospital to home" OR "care transition*" OR "transitional care" OR "continuity of care"<br>(“Maternal Health Services") OR (hospital* OR "health care facilit*" OR "healthcare facilit*" OR "health facilit*" OR "maternity ward*" OR "maternity unit*" OR "maternity care") OR (hospital* OR "health care facilit*" OR "healthcare facilit*" OR "health facilit*" OR "maternity ward*" OR "maternity unit*" OR "maternity care")	04102026	Academic Journals,<br>English Language<br>Publication: 2014-202<br>Full Text, Peer Reviewed, References available<br>Mothers<br>Publisher: Health Services Research, Social Sciences and Medicine
